# Engineered EGCG‐Containing Biomimetic Nanoassemblies as Effective Delivery Platform for Enhanced Cancer Therapy

**DOI:** 10.1002/advs.202105894

**Published:** 2022-03-25

**Authors:** Pengkai Wu, Haitian Zhang, Yin Yin, Meiling Sun, Shuai Mao, Huihui Chen, Yexuan Deng, Shuai Chen, Shuo Li, Beicheng Sun

**Affiliations:** ^1^ Department of Hepatobiliary Surgery Nanjing Drum Tower Hospital Clinical College of Nanjing Medical University Nanjing Jiangsu Province 210008 P. R. China; ^2^ Department of Hepatobiliary Surgery Nanjing Drum Tower Hospital The Affiliated Hospital of Nanjing University Medical School Nanjing Jiangsu Province 210008 P. R. China; ^3^ MOE Key Laboratory of Model Animal for Disease Study Department of Endocrinology Nanjing Drum Tower Hospital and Model Animal Research Center School of Medicine Nanjing University Nanjing 210008 P. R. China; ^4^ Department of Gastroenterology The First Affiliated Hospital of Nanjing Medical University Nanjing Jiangsu Province 210029 P. R. China

**Keywords:** biomimetic delivery, coordination, EGCG, fluorine, immunotherapy

## Abstract

Nano‐based immunotherapy of therapeutic biomolecules is attractive but tremendously hampered by the poor delivery efficiency. This study reports a novel delivery system of fluorinated‐coordinative‐epigallocatechin gallate (EGCG), referring as FEGCG/Zn, through the integration of fluorination and zinc ions (Zn^2+^) into EGCG. The robust therapeutics of FEGCG/Zn are measured in terms of the regulating effect on programmed cell death ligand 1 (PD‐L1), the effective delivery of diverse biomolecules, and the hitchhiking ability using living cells. Taking small interfering RNA of PD‐L1 (siPD‐L1) and erythrocytes as an example, the fabricated biomimetic system achieves excellent siPD‐L1 delivery and further improves siPD‐L1 accumulation in tumors. Finally, the combination of FEGCG/Zn and siPD‐L1 promotes antitumor immunotherapy through alleviation of T cells exhaustion by regulating PD‐L1 expression in tumor cells. The results demonstrate that FEGCG/Zn substantially regulates PD‐L1 expression and improves immune‐biomolecule delivery by forming biomimetic nanoassemblies, offering a versatile platform for cancer immunotherapy.

## Graphical abstract

The fluorinated‐coordinative polymer of FEGCG/Zn as an effective delivery platform is developed for immunotherapy with functions of PD‐L1 regulating, biomolecules delivery, and living‐cell hitchhiking. Based on these features, the constructed biomimetic nanoassemblies composed of FEGCG/Zn, siPD‐L1, and erythrocytes can increase their accumulation in tumors and alleviate T cells exhaustion, resulting in enhanced cancer immunotherapy.

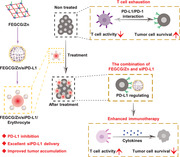

## 1 Introduction

Cancer presents a severe threat to human health, and immunotherapy has made remarkable progress in the disease treatment via mechanisms including the immune checkpoint blockade, for example, the inhibition of programmed cell death 1 (PD‐1)/programmed cell death ligand 1 (PD‐L1) pathway‐mediated tumor immune evasion.^[^ ^1^ ^]^ However, the efficacy of immunotherapy is often limited by insufficient T cells infiltration in solid tumors. A variety of biomolecule‐based therapeutic approaches such as chemotherapy,^[^ ^2^ ^]^ gene therapy,^[^ ^3^ ^]^ and peptide/protein therapy^[^ ^4^ ^]^ have been extensively and continuously developed to improve the outcome of immunotherapy. Despite all the improvements made by far, the delivery efficiency remains a bottleneck that limits therapeutic potency. Nanotechnology bears great potential for effective delivery of immune‐biomolecule, but it remains a central theme to develop promising drug delivery platform based on natural substances that possess good performance and safety.^[^ ^5^ ^]^

Epigallocatechin gallate (EGCG), a Food and Drug Administration (FDA) approved polyphenol from green tea, has recently attracted attentions in the fields of drug delivery and cancer therapy.^[^ ^6^ ^]^ The bioactivity of EGCG, including anti‐inflammatory, antioxidant, and immune‐modulatory activities, ensures its effectiveness in cancer treatment.^[^ ^7^ ^]^ Specifically, EGCG can act as an immune checkpoint inhibitor of PD‐L1 to enhance the antitumor immune response.^[^ ^8^ ^]^ Simultaneously, EGCG possesses rich hydroxyl groups and aromatic groups to provide different interactions, including hydrogen bond interactions, electrostatic interactions, coordination sites, and possible hydrophobic and *π*–*π* interactions for biomolecule complexation.^[^ ^6^, ^9^ ^]^ For example, EGCG exhibits strong binding affinity for peptides,^[^ ^10^ ^]^ proteins,^[^ ^11^ ^]^ and nucleic acids,^[^ ^12^ ^]^ which can facilitate the assembly of nanostructures and further attachment to cell surfaces by using metal–phenolic coordination to fabricate a biomimetic system for advanced cell‐based therapies.^[^ ^9^ ^]^ Despite these impressive profiles, the EGCG containing system‐related delivery efficacy still hinders the expansion of EGCG‐based materials.

A strategy that potentially optimizes the delivery efficacy described above is to synthesize a fluorinated‐coordinative‐EGCG, referring as FEGCG/Zn, through the integration of fluorination and zinc ions (Zn^2+^) into EGCG. Fluorination is a classic strategy to improve the stability and transfection efficacy of nanomaterials due to its lipophobic and hydrophobic features, which can result in stable phase separation with a high tendency.^[^ ^13^ ^]^ Another coordination strategy using zinc ions (Zn^2+^) confers nanomaterials great binding affinity with phosphate‐containing molecules (e.g., nucleic acids and phospholipids), anionic groups (e.g., carboxyl groups of peptide and protein), and imidazole and amine groups, which strengthens the interaction between the nanomaterial and cargo, and promotes the fusion of formed nanostructures with cell membranes.^[^ ^11^, ^14^ ^]^ In addition, FEGCG as a peroxalate polymer can be disrupted to release EGCG in the presence of reactive oxygen species (ROS) due to the high reactivity between peroxalate ester and H_2_O_2_.^[^ ^15^ ^]^ The pH‐responsive profile of metal polyphenol networks allows EGCG/Zn to release EGCG and Zn^2+^ in a pH‐dependent manner.^[^ ^16^ ^]^ Therefore, FEGCG/Zn represents a novel dual ROS/pH sensitive system with multiple functions, including inhibition of PD‐L1 expression, delivery of diverse biomolecules with robust efficiency, and hitchhiking ability by using living cells.

**Figure 1:**
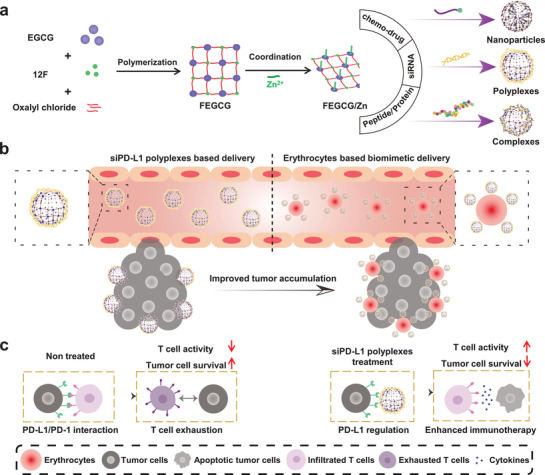
Schematic illustration represents the a) construction of the FEGCG/Zn system and self‐assembled nanoassemblies with chemo‐drug, siRNA, and peptide/protein, b) erythrocyte‐based biomimetic delivery to improve the tumor accumulation of siRNA polyplexes, and c) mechanism of FEGCG/Zn/siPD‐L1 polyplexes in enhanced cancer immunotherapy.

To investigate the feasibility of FEGCG/Zn as an effective delivery platform, the nanoassemblies‐forming features were evaluated in chemical drugs (sorafenib [Sor], gemcitabine [Gem], and doxorubicin [DOX]), nucleic acids (small interfering RNA [siRNA]), peptides (melittin [MPI]), and proteins (bovine serum albumin [BSA]) by parameters including drug loading, stability, cargo release, and cytosolic delivery efficiency (**Figure** **1**a). Considering well‐documented efficacy of PD‐L1‐based immunotherapy and prolonged circulation time with erythrocyte‐based drug delivery,^[^ ^17^ ^]^ siPD‐L1 and erythrocytes were selected to validate the potency of the fabricated biomimetic delivery system. Our results showed that not only the erythrocytes part of the system could improve the tumor accumulation of siPD‐L1, but also the released FEGCG/Zn and siPD‐L1 could alleviate T cells exhaustion by blocking PD‐1/PD‐L1 engagement, resulting in the enhanced efficacy of anti‐PD‐L1 immunotherapy (Figure 1b‐c).

## 2 Results and Discussion

### 2.1 Synthesis, Characterization, and Bioactivity Evaluation of FEGCG/Zn

The fluoropolymer of FEGCG was first synthesized through the reaction of fluorinated compound (12F) and EGCG with the ROS‐sensitive linker of oxalyl chloride (Figure S1, Supporting Information). The structure of FEGCG was demonstrated by ^1^H‐NMR and ^19^F‐NMR, and its average molecular weight was ≈7000 Da, as determined by gel permeation chromatography (Figure S2, Supporting Information). The conjugation ratios of EGCG and 12F in FEGCG were 0.41 and 0.59, respectively. The fluorinated‐coordinative polymer of FEGCG/Zn was then obtained through the coordination with Zn^2+^ at the FEGCG/Zn molar ratio of 1:2. The successful coordination of Zn^2+^ and FEGCG was confirmed by ICP‐MS, and the content of Zn^2+^ was 21.9 mg/100 mg FEGCG. Subsequently, their bioactivities were evaluated by measuring the free radical scavenging, cytotoxicity, apoptosis, PD‐L1 inhibiting and migration abilities compared with those of the parental EGCG. As shown in Figure S3a (Supporting Information), neither fluorination nor Zn^2+^ coordination significantly affected the in vitro free radical scavenging ability of EGCG. Meanwhile, its killing effects on toxicity and apoptosis and the inhibition of PD‐L1 expression and cell migration were enhanced after functionalization (Figure S3b–e, Supporting Information). In addition, all of them exhibited negligible level of hemolysis, suggestive of excellent biocompatibility during blood circulation (Figure S4, Supporting Information). Therefore, FEGCG/Zn represented a promising system for cancer therapy because of its improved bioactivities compared with EGCG, which might be attributed to its increased affinity with cell membranes.

**Figure 2:**
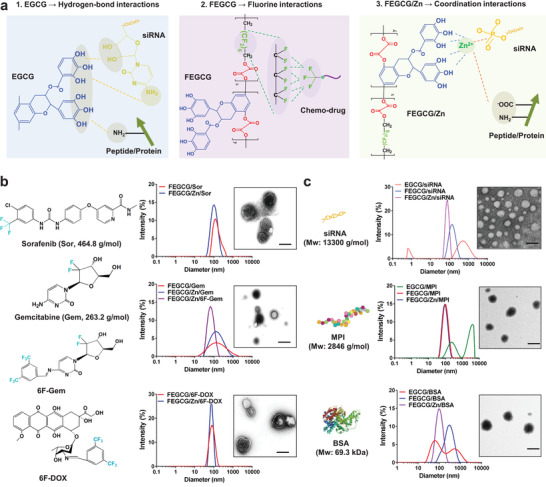
a) Schematic illustration reveals the driving forces provided by FEGCG/Zn for delivery of diverse biomolecules. b) Size distribution and TEM images of Sor NPs, Gem/6F‐Gem NPs, and 6F‐DOX NPs. c) Size distribution and TEM images of siRNA polyplexes, MPI complexes, and BSA complexes. Scale bar = 100 nm.

The superior properties of FEGCG/Zn as a delivery platform, including high drug loading, sensitive release, satisfactory stability and efficient cytosolic delivery, are dependent on integrated driving forces provided by EGCG, fluorine and Zn^2+^. Primarily, hydrogen‐bond interactions, fluorine interactions, and coordination interactions of FEGCG/Zn contribute to the diverse biomolecule delivery (**Figure** **2**a). First, chemo‐drug was tested for nanoparticles (NPs)‐forming ability. Sor (three fluorine atoms, 464.8 g mol^−1^) and Gem (two fluorine atoms, 263.2 g mol^−1^) were chosen as the model of fluorinated chemo‐drugs as ≈30% of the marketed drugs contained one or more fluorine atoms in their structure.^[^ ^18^ ^]^ The carriers were mixed with the drug to obtain the NPs through a nanoprecipitation method.^[^ ^19^ ^]^ The driving force from EGCG could not provide interactions for Sor/Gem NPs formation. Obviously, the fluorine interactions between FEGCG and Sor/Gem could promote the NPs formation with the particle size of ≈122 and ≈142 nm, respectively. The introduced coordination interactions from Zn^2+^ in FEGCG/Zn further decreased the size distribution of Sor NPs and Gem NPs with size of ≈110 and ≈122 nm, respectively (Figure 2b). This phenomenon may be due to the increased affinity between Zn^2+^ and the amino group of drugs.

To further confirm the above results and extend the current strategy to a wider range of chemo‐drugs, the prodrug strategy was used to demonstrate the advantages of fluorine interactions in mediating drug delivery. The pH‐sensitive prodrugs of DOX (543.5 g mol^−1^) and Gem, referred to as 6F‐DOX and 6F‐Gem, respectively, were synthesized through the Schiff base reaction with 3,5‐bis(trifluoromethyl) benzaldehyde (6F‐Ben).^[^ ^20^ ^]^ Their structures were confirmed by ^1^H‐NMR and ^19^F‐NMR (Figures S5 and S6, Supporting Information). The size distribution of FEGCG/Zn/6F‐Gem NPs was more uniform and smaller than that of FEGCG/Zn/Gem NPs (68 nm vs 122 nm). As expected, the NPs could be self‐assembled between FEGCG or FEGCG/Zn and 6F‐DOX, and their particle size was ≈110 nm. Transmission electron microscope (TEM) results confirmed the successful formation of FEGCG/Zn/Sor NPs, FEGCG/Zn/6F‐Gem NPs, and FEGCG/Zn/6F‐DOX NPs, and their zeta potential was 22, 10, and 44 mV, respectively (Figure 2b and Figure S7a, Supporting Information).

Next, siRNA (13 300 g mol^−1^), MPI (2846 g mol^−1^), and BSA (69.3 kDa) were selected as the prototype for nucleic acids, peptides, and proteins, respectively, for validating the superiority of FEGCG/Zn‐mediated fluorine and coordination interactions when compared with EGCG/Zn‐mediated hydrogen‐bond interactions. Although fluorine or Zn^2+^ alone has been used to improve the delivery of siRNA, few studies have showed the synergistic effect of the two combined.^[^ ^13^, ^14^, ^21^ ^]^ The siRNA polyplexes were prepared by gently mixing the carrier and siRNA. As shown in Figure 2c, FEGCG/Zn achieved smaller and more uniform size distribution, followed by FEGCG and EGCG (78 nm vs 142 nm vs 531 nm).

Similar to siRNA delivery as described above, no platform has been reported to combine fluorine and Zn^2+^ modification for peptide/protein delivery.^[11,13a]^ As shown in Figure 2c, the complexes were formed by the carrier and MPI/BSA using a gentle mixing method. Compared to EGCG complexes, the size distribution of FEGCG/MPI complexes and FEGCG/Zn/MPI complexes was more uniform at around 91 nm. Consistently, better uniformed complexation was also observed for FEGCG/Zn/BSA complexes compared to FEGCG/BSA and EGCG/BSA complexes, of which the size distribution was 106, 295, and 531 nm, respectively. This suggested that the fluorination may enhance the binding affinity of EGCG, and the coordination capability of Zn^2+^ further promoted its affinity with peptide/protein, in particular contains multiple carboxyl and amine groups. In support of this notion, TEM images further confirmed the successful formation of FEGCG/Zn/siRNA polyplexes, FEGCG/Zn/MPI complexes and FEGCG/Zn/BSA complexes, of which the zeta potential was −4.5, 39, and −5.3 mV, respectively (Figure S7b, Supporting Information). These results demonstrated that FEGCG/Zn system has the potential to deliver biomolecules ranging from small molecule drugs to larger macromolecules.

### 2.2 Drug Loading, Stability, Sensitive Release, and Cytosolic Delivery of FEGCG/Zn

**Figure 3:**
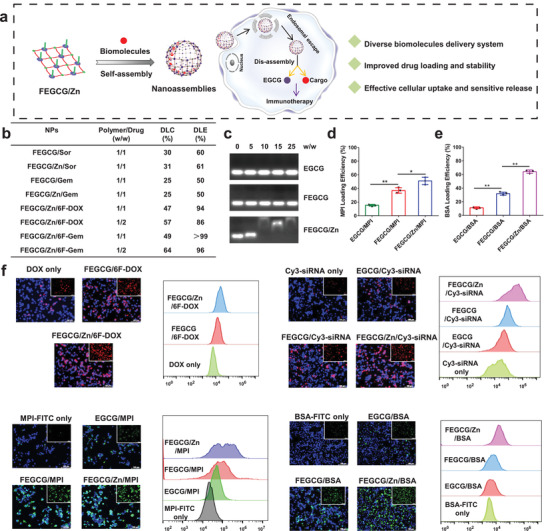
a) Schematic illustration of FEGCG/Zn based nanoassemblies and their advantages in biomolecule delivery in vitro. b) DLC and DLE of different chemo‐drugs in FEGCG or FEGCG/Zn NPs. c) siRNA loading efficiency determined by agarose gel electrophoresis. d,e) MPI or BSA loading efficiency determined by FITC fluorescence quenching assay (*n* = 3). The cytosolic delivery efficiency of DOX NPs, Cy3‐siRNA polyplexes, FITC‐MPI complexes, and FITC‐BSA complexes determined by confocal microscopy and flow cytometry. Scale bar = 100 µm. f) Data are presented as the means ± SD. Error bars represent the standard deviations of three separate measurements. *P < 0.05, **P < 0.01 by one‐way analysis of variance (ANOVA) followed by Turkey's multiple comparisons.

All the features described above are essential for the design of NPs based drug delivery system, in particular to circumvent challenges such as inefficient chemo‐drugs loading,^[^ ^22^ ^]^ instability and low delivery efficiency for macromolecules,^[^ ^23^ ^]^ and uncontrolled drug release. Another potential advantage of EGCG as delivery carrier is that it minimizes the toxicity and immunogenicity caused by excessive nanomaterial usage. Therefore, FEGCG/Zn represents a new platform to play the functions of biomolecules delivery and immunotherapy (**Figure** **3**a). As shown in Figure 3b, the drug loading capacity (DLC) and drug loading efficiency (DLE) of Sor in FEGCG NPs and FEGCG/Zn NPs were nearly the same with ≈30% and ≈60% at w/w (polymer/drug) = 1/1, respectively. Together, the DLC and DLE of Gem in FEGCG NPs and FEGCG/Zn NPs were also the same with ≈25% and j50% at w/w (polymer/drug) = 1/1, respectively. These results indicated that more fluorine atoms in chemo‐drugs represented stronger fluorine interactions, attributed to high DLC and DLE. In contrast, the apparent drug precipitates appeared after mixing EGCG and Sor/Gem. Our prodrug strategy further demonstrated the above results, which were reflected in the superior DLC with ≈50% and DLE with ≈90% at different w/w ratios of 6F‐DOX NPs and 6F‐Gem NPs. For siRNA loading, the agarose gel electrophoresis experiment revealed that FEGCG/Zn could condense siRNA at w/w 10, whereas FEGCG and EGCG did not condense siRNA even at w/w 25 (Figure 3c). Using Cy5‐labeled siPD‐L1 as a tool, FEGCG/Zn and FEGCG showed maximum fluorescence quenching compared with EGCG (Figure S7c, Supporting Information). For MPI/BSA loading, their loading efficiency was determined using a fluorescence quenching method. As shown in Figure 3d,e (Figure S8a,b, Supporting Information), the amount of MPI or BSA binding to EGCG in deionized water was less than 20%, whereas the loading efficiency of FEGCG and FEGCG/Zn was significantly increased to 37% of MPI and 51% of BSA, 31% of MPI and 64% of BSA, respectively.

To interrogate the stability profile, the size change of FEGCG/Zn/6F‐Gem, FEGCG/Zn/Sor and FEGCG/Zn/6F‐DOX NPs was monitored in PBS media. As shown in Figure S9a (Supporting Information), all tested NPs exhibited good stability in PBS medium since no significant size changes were observed even after incubation for up to 24 h. The RNase stability results revealed that FEGCG/Zn, FEGCG and ECGC could maintain Cy5 siRNA activity at 60%, 36%, and 12% under RNase enzyme incubation conditions, respectively (Figure S9b, Supporting Information). Together, the ability of FEGCG/Zn complexes against salt solutions was stronger than that of FEGCG complexes, followed by EGCG complexes, which suggests the benefits of combined fluorine and Zn^2+^ on nanostructures stability (Figure S9c,d, Supporting Information).

The nanostructures formed by FEGCG/Zn and biomolecules are both sensitive to pH and ROS, and their hydrolysis profile was conducted by coincubation with H_2_O_2_ and different pH values. Taking FEGCG/Zn/6F‐DOX NPs as an example, ≈64% drug release could be observed when the NPs were treated with both H_2_O_2_ and pH 5.0 compared with ≈42% (H_2_O_2_), ≈27% (pH 5.0), or ≈12% (pH 7.4) (Figure S10a, Supporting Information). Subsequently, ROS/pH triggered siRNA release was evaluated by incubating FEGCG/Zn polyplexes with different media. The results showed that ≈42.0% Cy5‐siRNA could be released from the polyplexes treated with both H_2_O_2_ and pH 5.0 compared with either condition only (≈10.1% for H_2_O_2_ and ≈1.8% for pH 5.0) (Figure S10b, Supporting Information). Together, ROS/pH triggered MPI/BSA release was also evaluated by incubating FEGCG/Zn complexes with different media. The results showed that ≈26.0% MPI‐FITC and ≈42.0% BSA‐FITC could be released from the related complexes treated with both H_2_O_2_ and pH 5.0, respectively (Figure S10c,d, Supporting Information). The cytosolic delivery efficiency of various FEGCG/Zn nanostructures was examined by detecting the fluorescence of DOX, Cy3‐siRNA, FITC‐MPI, and FITC‐BSA in Hep1‐6 cells, a liver cancer cell line. As shown in Figure 3f, confocal microscopy and flow cytometry experiments demonstrated that FEGCG/Zn with fluorine and coordination interactions achieved the highest cellular uptake, followed by FEGCG and EGCG. The superior delivery efficiency of FEGCG/Zn was further confirmed in MHCC‐97H cells (liver cancer) and 4T1 cells (breast cancer) (Figure S11, Supporting Information). These results indicated that the integration of fluorination and Zn^2+^ on EGCG was a feasible and effective strategy to overcome the limitations in biomolecule delivery.

### 2.3 Biodistribution of Erythrocyte‐Mediated FEGCG/Zn/Cy5‐siRNA Biomimetic System

**Figure 4:**
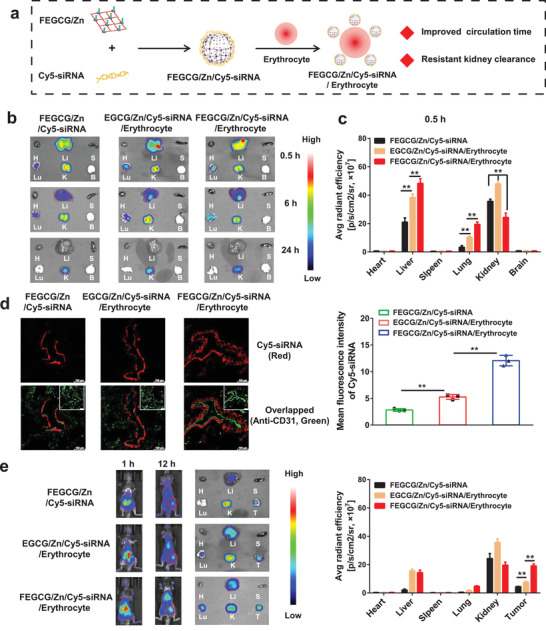
a) Schematic illustration in engineering of FEGCG/Zn/siRNA/erythrocyte biomimetic system and its advantages in siRNA delivery in vivo. b,c) Biodistribution and quantitative analysis of FEGCG/Zn/Cy5‐siRNA, EGCG/Zn/Cy5‐siRNA/erythrocyte, and FEGCG/Zn/Cy5‐siRNA/erythrocyte 0.5 h after i.v. administration in healthy mice (*n* = 3). d) Confocal images and quantitative analysis of lung tissues 0.5 h after i.v. administration of different administrations (*n* = 3). Red fluorescence represents Cy5‐siRNA, and green fluorescence represents vascular capillaries stained with Alexa Fluor 488‐conjugated anti‐CD31 antibody. Scale bar = 100 µm. e) Biodistribution and quantitative analysis of FEGCG/Zn/Cy5‐siRNA, EGCG/Zn/Cy5‐siRNA/erythrocyte, and FEGCG/Zn/Cy5‐siRNA/erythrocyte 1 and 12 h after i.v. administration in Hep1‐6 tumor‐bearing mice (*n* = 3). The abbreviations are shown as H of heart, Li of liver, S of spleen, Lu of lung, K of kidney, B of brain, and T of tumor. Data are presented as the means ± SD. Error bars represent the standard deviations of three separate measurements. *P < 0.05, **P < 0.01 by one‐way ANOVA analysis followed by Turkey's multiple comparisons.

To investigate the function of FEGCG/Zn in hitchhiking ability by using living cells, Cy5‐labeled siPD‐L1 (Cy5‐siRNA) as the model of biomolecules and erythrocytes as the model of living cells were selected to fabricate the erythrocyte‐based biomimetic system with a mixture of FEGCG/Zn/siRNA polyplexes and erythrocytes. Considering the enhanced stability of fluorine modification and prolonged circulation time of erythrocytes, we analyzed the effect of fluorine and erythrocyte‐based delivery on the organ distribution of FEGCG/Zn/Cy5‐siRNA/erythrocyte with FEGCG/Zn/Cy5‐siRNA polyplexes (fluorination alone) and EGCG/Zn/Cy5‐siRNA/erythrocyte system (erythrocyte alone) as controls (**Figure** **4**a). It should be noted that FEGCG/Zn/Cy5‐siRNA/erythrocyte system needs to be prepared in vitro rather than in vivo when considering the nonspecific adsorption of FEGCG/Zn/Cy5‐siRNA to other cells, such as T cells, monocytes, macrophages, dendritic cells, and NK cells. Confocal images revealed the successful assembly of FEGCG/Zn/siRNA polyplexes on erythrocytes because of the similar spatial distribution of erythrocytes and FEGCG/Zn/siRNA/erythrocyte system (Figure S12, Supporting Information). Biodistribution results in healthy mice revealed that major signal intensities appeared in liver, lung, and kidney at 0.5 h after the administration of FEGCG/Zn/Cy5‐siRNA polyplexes without erythrocytes. EGCG/Zn/Cy5‐siRNA/erythrocyte without fluorination system were found to exhibit stronger intensities in the above organs than FEGCG/Zn/Cy5‐siRNA polyplexes. Moreover, FEGCG/Zn/Cy5‐siRNA/erythrocyte system with the combination of fluorination and erythrocytes further enhanced the liver and lung accumulation but decreased the accumulation in the kidney, which suggested potential benefits of combined erythrocytes and fluorination on prolonged circulation time and resistant kidney clearance. As time progressed, a similar trend could still be observed at 6 and 24 h post‐injection (Figure 4b,c and Figure S13, Supporting Information).

We also analyzed lung tissues to observe the distribution of FEGCG/Zn/Cy5‐siRNA/erythrocyte system within microstructures of the lung as the lung is a hard‐to‐reach organ compared with liver for i.v. injected nanostructures. As shown in Figure 4d, FEGCG/Zn/Cy5‐siRNA/erythrocyte system was able to deliver large amounts of Cy5‐siRNA, which was highly surrounded by anti‐CD31 marked capillary vascular microstructures compared with the other two groups. These results indicated that the cargo of Cy5‐siRNA could be protected by FEGCG/Zn and extended the systemic circulation through attachment with erythrocytes in vivo. Finally, the distribution study of FEGCG/Zn/Cy5‐siRNA/erythrocyte system was re‐performed on Hep1‐6 tumor‐bearing mice, and the fluorescence intensity of isolated tumors was analyzed at 12 h postinjection. The tumor uptake in FEGCG/Zn/Cy5‐siRNA/erythrocyte system was ≈2‐fold than that in EGCG/Zn/Cy5‐siRNA/erythrocyte and ≈4‐fold than that in FEGCG/Zn/Cy5‐siRNA polyplexes (Figure 4e). The distribution results of other organs were similar to those of healthy mice. These results revealed that FEGCG/Zn could represent a novel system to further improve the potency of loaded cargos by using the hitchhiking ability of living cells.

### 2.4 Enhanced Immunotherapy of siPD‐L1 by FEGCG/Zn and Erythrocyte

**Figure 5:**
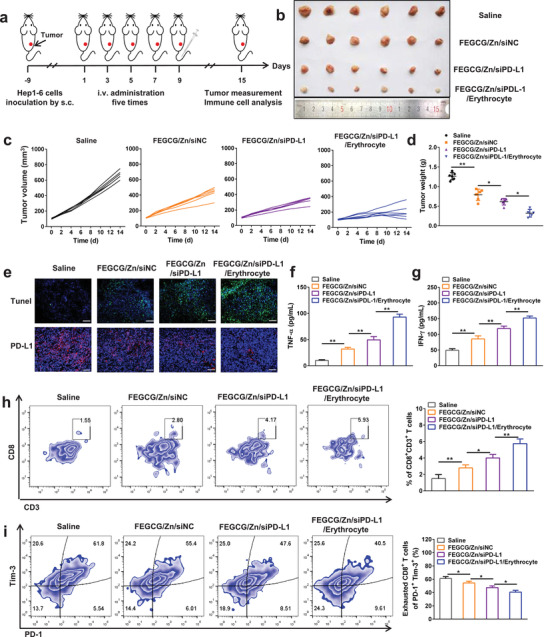
Antitumor immunotherapy and immune activation in vivo. a) Schematic of the treatment regimen. b) Representative photographs of collected tumor tissues, c) the tumor growth curves of individual mice, and d) the tumor weight after different treatments of saline, FEGCG/Zn/siNC, FEGCG/Zn/siPD‐L1, and FEGCG/Zn/siPD‐L1/erythrocyte (*n* = 6). e) Representative images of TUNEL and PD‐L1 immunofluorescence staining in tumor tissues. Scale bar = 100 nm. f,g) The levels of TNF‐*α* and IFN‐*γ* in plasma (*n* = 6). Flow cytometry assay and quantitative analysis of CD8^+^CD3^+^T cells in h) CD45^+^ cells and i) exhausted CD8^+^ T cells in lymphocytes (*n* = 5). Data are presented as the means ± SD. Error bars represent the standard deviations of at least three separate measurements. *P < 0.05, **P < 0.01 by one‐way ANOVA analysis followed by Turkey's multiple comparisons.

Finally, we examined the combined immunotherapy efficacy of FEGCG/Zn and siPD‐L1 using an erythrocyte‐based delivery system, and then explored the potential antitumor mechanism by focusing on infiltrated CD8^+^ T cells and exhausted CD8^+^ T cells. A Hep1‐6 xenograft model was established to evaluate the antitumor efficacy of FEGCG/Zn/siPD‐L1/erythrocyte system with FEGCG/Zn/siNC (scrambled siRNA) and FEGCG/Zn/siPD‐L1 treatments as controls. The treatment regimen was performed according to the scheme of **Figure** **5**a. Representative photographs of tumor tissues and changes in tumor volume and tumor weight were shown in Figure 5b–d. The tumor progression was significantly inhibited in each treatment group of FEGCG/Zn/siNC, FEGCG/Zn/siPD‐L1 and FEGCG/Zn/siPD‐L1/erythrocyte compared to the saline group. Among them, FEGCG/Zn/siNC exhibited a significant tumor suppression effect, likely due to the reported antitumor activity of EGCG.^[^ ^24^ ^]^ After combination with siPD‐L1, the tumor weight after FEGCG/Zn/siPD‐L1 treatment was further reduced. Particularly, after attachment to erythrocytes, FEGCG/Zn/siPD‐L1/erythrocyte treatment achieved the best antitumor effect, largely due to the enhanced tumor accumulation demonstrated in the distribution study. The body weight of these four groups showed no significant change during the treatment period (Figure S14a, Supporting Information). In addition, H&E staining results showed that all treatments especially FEGCG/Zn/siPD‐L1/erythrocyte group induced clear necrotic lesions (Figure S15, Supporting Information). TUNEL and PD‐L1 immunofluorescence staining results showed that the main treatments increased the apoptosis of tumor cells and decreased the expression of PD‐L1 in tumor tissues (Figure 5e and Figure S14b, Supporting Information).

To investigate the potential immune activation, plasma levels of tumor necrosis factor‐*α* (TNF‐*α*) and interferon‐*γ* (IFN‐*γ*) were examined,^[^ ^25^ ^]^ which were significantly upregulated after the combined treatment of FEGCG/Zn, siPD‐L1 and erythrocytes (Figure 5f,g). The lymphocytes were then isolated from tumors and the activation of infiltrated CD8^+^ T cells (CD8^+^CD3^+^) was determined. After the FEGCG/Zn/siPD‐L1/erythrocyte treatment, the ratio of CD8^+^CD3^+^ T cells was 15.3%, which was higher than that of the FEGCG/Zn/siPD‐L1 treatment (10.3%), the FEGCG/Zn/siNC treatment (7.6%), and the saline group (5.2%) (Figure 5h). One of the possible mechanisms is that the T cells activity is enhanced through the alleviation of T cell exhaustion.^[^ ^26^ ^]^ As shown in Figure 5i, the count of exhausted CD8^+^ T cells (PD1^+^Tim3^+^) decreased from 61.8% in the saline group to 55.4% and 47.6% after FEGCG/Zn/siNC treatment or FEGCG/Zn/siPD‐L1 treatment, respectively. As anticipated, the ratio of exhausted CD8^+^ T cells in the FEGCG/Zn/siPD‐L1/erythrocyte group was further reduced to 40.5% through biomimetic delivery of erythrocytes.

### 2.5 Biosafety Evaluation of FEGCG/Zn Biomimetic System

The biosafety of FEGCG/Zn/siNC polyplexes, FEGCG/Zn/siPD‐L1 polyplexes, and FEGCG/Zn/siPD‐L1/erythrocyte system was also evaluated in healthy mice. The major organs, including heart, liver, spleen, lung, and kidney, were harvested for H&E analysis. Blood samples were collected for hepatic function biomarkers of alanine aminotransferase (ALT) and aspartate aminotransferase (AST) determination, and the blood analysis of white blood cells (WBCs), lymphocytes, neutrophils, monocytes, and platelets. Compared with the saline group, there were no notable change in ALT and AST levels, physiological difference, and the number of blood cells in each treatment group (Figures S16 and S17, Supporting Information). All of these data emphasized the inspiring therapeutic effects of FEGCG/Zn as a functional system in cancer immunotherapies.

## 3 Conclusion

In summary, we have engineered a novel delivery system of FEGCG/Zn that can simultaneously regulate PD‐L1 expression and deliver a variety of biomolecules. Taking advantage of the hitchhiking ability of living cells, the fabricated biomimetic system can further enhance the potency of delivered cargos. The multiple driving forces provided by FEGCG/Zn ensure superior profiles, including high drug loading, sensitive release, satisfactory stability, and efficient cytosolic delivery. This was confirmed using Sor, Gem, and DOX as the model of chemical drugs, siRNA as the model of nucleic acids, MPI as the model of peptides, and BSA as the model of proteins, which all revealed that FEGCG/Zn could act as an effective delivery platform compared to EGCG. Enhanced anti‐PD‐L1 immunotherapy was realized by fabricating FEGCG/Zn/siPD‐L1/erythrocyte biomimetic system. The erythrocytes part could improve siPD‐L1 accumulation in tumors, and the combination of FEGCG/Zn and siPD‐L1 could boost tumor‐killing of T cells likely due to blocking PD‐L1/PD‐1‐mediated inhibitory pathway that dampens the activity of infiltrated CD8^+^ T cells. The results presented in this study demonstrate that FEGCG/Zn can act as both PD‐L1 inhibitor and carrier for immune‐biomolecule delivery and cell‐based delivery, and has potential as an effective delivery platform for enhanced cancer therapy.

## 4 Experimental Section

### Materials

(−)‐epigallocatechin‐3‐O‐gallate (EGCG), dimethylformamide (DMF), dichloromethane (DCM), and methanol (CH_3_OH) were purchased from Energy Chemical (Shanghai, China). 2,2‐Diphenyl‐1‐picrylhydrazyl (DPPH) and 2,2,3,3,4,4,5,5,6,6,7,7‐dodecafluoro‐1,8‐octanediol (12F) were purchased from Macklin Biochemical Co., Ltd. (Shanghai, China). Sorafenib, gemcitabine, and doxorubicin hydrochloride were purchased from MedChemExpress (New Jersey, USA). Oxalyl chloride solution was purchased from Aladdin Bio‐Chem Technology Co., Ltd. (Shanghai, China). FAM‐, Cy3‐, and Cy5‐labeled siRNA and siNC (sense strand, 5′‐UUC UCC GAA CGU GUC ACG UTT‐3′), siPD‐L1 (sense strand, 5′‐ AGG AUG GUU CUU AGA CUU UTT‐3′) were purchased from GenePharma Co., Ltd. (Shanghai, China). RNase enzyme and DAPI were purchased from Beyotime Biotechnology (Shanghai, China). Mouse TNF‐*α* and IFN‐*γ* ELISA kit were purchased from Wuhan Boster Biological Technology Co. Ltd. All other reagents were obtained from Beyotime Biotechnology Co., Ltd. (Shanghai, China) unless otherwise stated.

### Synthesis of FEGCG and FEGCG/Zn

The fluoropolymers of FEGCG were synthesized by gently mixing 12F, EGCG, and oxalyl chloride. Briefly, the DCM solutions of EGCG (275 mg, 0.6 × 10^−3^ m) and oxalyl chloride (127 mg, 1.0 × 10^−3^ m) were slowly mixing with the DMF solution of 12F (145 mg, 0.4 × 10^−3^ m), and the reacted solution was kept under nitrogen atmosphere for 6 h. The final product was purified by evaporating the solvent, washing with diethyl ether three times and dialyzing against deionized water (molecular weight cut off 1.0 kDa). FEGCG was characterized by ^1^H‐NMR, ^19^F‐NMR (Bruker 600 MHz), and gel permeation chromatography (Agilent 1260 Infinity LC system). The synthesized FEGCG were then dissolved in methanol and reacted with Zn (NO_3_)_2_·6H_2_O at Zn^2+^/FEGCG molar ratio of 2:1. The reactions were stirred for 6 h at room temperature, and evaporated and dialyzed to obtain FEGCG/Zn. The amount of Zn^2+^ in FEGCG/Zn was detected by inductively coupled plasma mass spectrometry (ICP‐MS).

### Bioactivity Studies

It should be noted that the concentration of FEGCG in this study was expressed as equivalent EGCG concentration, excluding 12F content. Free radical scavenging ability of EGCG, FEGCG, and FEGCG/Zn was evaluated using a DPPH method. Briefly, the ethanol solution of DPPH was prepared at the concentration of 1 mg mL^−1^, and further incubated with the above carriers (10 µg mL^−1^) for 30 min at room temperature. The remaining radical of the solution was detected at absorbance (*A*) of 515 nm and calculated according to the following formula (ethanol blank solution and DPPH ethanol solution served as negative and positive controls, respectively)

(1) $$\begin{array}{c} \mathrm{Remaining}\;\mathrm{radical}\;\left(\%\right)=A_{\mathrm{DPPH}}-A_{\mathrm{treatments}}/A_{\mathrm{DPPH}}-A_{\mathrm{blank}\;\mathrm{ethanol}}\times 100 \end{array}$$

The toxicity or apoptosis studies of EGCG, FEGCG, and FEGCG/Zn were evaluated by a standard CCK8 or Annexin V‐FITC method in Hep1‐6 cells. Briefly, the cells were seeded in 96‐ or 12‐well plates until ≈80% density. For toxicity, the cells were incubated with different carriers of 0.1, 10, 30, 50, 70, and 100 µg mL^−1^ for 24 h, and then a standard CCK8 assay was performed to determine their cytotoxicity. For apoptosis, the cells were incubated with 10 µg mL^−1^ of different carrier for 24 h, and then the assay was performed according to Annexin V‐FITC Apoptosis Detection Kit (BD Biosciences).

The cell migration ability of EGCG, FEGCG, and FEGCG/Zn was evaluated using a transwell model. Briefly, Hep1‐6 cells were added to the upper chamber with 10 µg mL^−1^ of different carrier. The culture medium containing 20% FBS was added to the lower chamber, and then the cells were cultured for 24 h. The upper cells of filter were removed, and the lower cells were fixed, stained and imaged.

The PD‐L1 silencing ability of EGCG, FEGCG and FEGCG/Zn was evaluated using a standard Western blot assay. Briefly, Hep1‐6 cells were pretreated with IFN‐*γ* (5 ng mL^−1^) and further treat with different carrier (10 µg mL^−1^) for 24 h. The cells were washed and the related proteins were isolated according to a standard procedure. The protein concentrations and levels were determined by a BCA Protein Assay Kit and Western blot, respectively.

For the hemolysis assay, erythrocytes were separated from the blood of C57BL/6 female mice, and their density was adjusted to 5 × 10^7^ mL^−1^. After that, the erythrocytes were incubated with PBS, EGCG (2 mg mL^−1^), FEGCG (2 mg mL^−1^), FEGCG/Zn (2 mg mL^−1^), and 1% Triton at 37 °C for 4 h. After incubation, samples were centrifuged to capture the image. The hemoglobin release was detected by a multimode reader at 540 nm.

### Drug Loading and Encapsulation Efficiency of Chemo‐Drug‐Loaded NPs

The chemo‐drug loaded NPs were obtained by a typical mixing method. Briefly, EGCG or FEGCG or FEGCG/Zn was dissolved in DMSO with the chemo‐drug, and then the above solution was stirred at room temperature for 1 h. The NPs were obtained by dialysis (MWCO 1000 Da) against distilled water. To determine the drug content in the NPs, they were centrifuged and collected for the measurement using a UV–vis spectrometer. Loading of Sor, Gem (6F‐Ben‐Gem), and DOX (6F‐DOX) was similarly processed, and drug loading was quantified by UV absorption at 285, 269, and 480 nm, respectively. The drug encapsulation efficiency and drug loading capacity were determined by dissolving NPs in DMF and measuring the related absorbance. The calculated formulas as follows

(2) $$\begin{array}{c} \text{The}\;\text{drug}\;\text{encapsulation}\;\text{efficiency}=\frac{\text{weight}\;\text{of}\;\text{drug}\;\text{in}\;\text{the}\;\text{NPs}}{\text{weight}\;\text{of}\;\text{total}\;\text{drug}}\times 100\% \end{array}$$

(3) $$\begin{array}{c} \begin{array}{c} \text{The}\;\text{drug}\;\text{loading}\;\text{capacity}=\frac{\text{weight}\;\text{of}\;\text{drug}\;\text{in}\;\text{the}\;\text{NPs}}{\text{weight}\;\text{of}\;\text{polymer}\;\text{and}\;\text{drug}\;\text{in}\;\text{the}\;\text{NPs}}\times 100\% \end{array} \end{array}$$

### Synthesis of 6F‐DOX and 6F‐Gem Prodrugs

The DOX or Gem prodrug was synthesized by the Schiff base reaction between the amino groups (—NH_2_) of DOX/Gem and the aldehyde groups (—CHO) of 6F‐Ben. Briefly, DOX (543.5 mg, 1 × 10^−3^ m)/Gem (263.2 mg, 1 × 10^−3^ m) and 6F‐Ben (242.1 mg, 1 × 10^−3^ m) were codissolved in 5 mL dry CH_3_OH with slight trace acetic acid as catalyzer, and the reaction was kept at room temperature for 24 h. The prodrug was purified by the column chromatography method (CH_2_Cl_2_:CH_3_OH, 10:1), and their structure was characterized by ^1^H‐NMR and ^19^F‐NMR (Bruker 600 MHz).

### Characterization of Chemo‐Drug‐Loaded NPs

The particle size and zeta potential of chemo‐drug loaded NPs were determined by dynamic light scattering (DLS) (Malvern Instruments Ltd., UK). The colloidal stability of FEGCG/Zn/6F‐Gem, FEGCG/Zn/Sor, and FEGCG/Zn/6F‐DOX NPs was performed by incubating them with PBS (pH 7.4) for 24 h, and their particle size was recorded. FEGCG/Zn/6F‐DOX NPs as a model was used to evaluate the ROS/pH triggered drug release. The nanoparticles were incubated with or without pH 5.0 PBS, 100 × 10^−6^ m H_2_O_2_ and both pH 5.0 and 100 × 10^−6^ m H_2_O_2_ for 36 h, respectively, and the DOX concentration was determined at the indicated time point.

### Preparation and Characterization of siRNA‐Loaded Polyplexes

The polyplexes were prepared by mixing the carrier and siRNA solution at desired w/w ratios for 30 min before use. The particle size and zeta potential of siRNA polyplexes were determined by DLS. The siRNA condense ability of EGCG, FEGCG, and FEGCG/Zn was determined by agarose gel electrophoresis at 100 V for 15 min with polyplexes prepared at w/w 0, 5, 10, 15, 25 (siRNA concentrations = 40 µg mL^−1^). The Cy5‐siRNA fluorescence quenching of EGCG, FEGCG, and FEGCG/Zn was characterized by incubating the polyplexes prepared at w/w 25 (siRNA concentrations = 266 µg mL^−1^) for 30 min. The Cy5‐siRNA fluorescence was determined by multimode reader (excitation at 560 nm, emission at 620–740 nm). The RNase stability of EGCG, FEGCG, and FEGCG/Zn polyplexes was evaluated by incubating them with RNase at 37 °C for 2 h, and the Cy5‐siRNA fluorescence was determined by multimode reader. The ROS/pH triggered siRNA release from FEGCG/Zn polyplexes was evaluated by incubating them in medium with or without pH 5.0 PBS, 100 × 10^−6^ m H_2_O_2_ and both pH 5.0 and 100 × 10^−6^ m mM H_2_O_2_ for 24 h, respectively, and the Cy5‐siRNA fluorescence was determined by multimode reader.

### Preparation and Characterization of Peptide/Protein‐Loaded Complexes

The complexes were prepared by complexing the carrier with MPI/BSA at w/w = 1 for 30 min (MPI/BSA concentrations = 10 µg mL^−1^). The particle size and zeta potential of MPI/BSA complexes were determined by DLS. The loading efficiency of MPI/BSA was determined using a FITC fluorescence quenching assay (excitation at 450 nm, emission at 480–600 nm). The calculated formulas as follows

(4) $$\text{The}\;\text{loading}\;\text{efficiency}=\frac{\text{The}\;\mathrm{maximum}\;\text{fluorescence}\;\text{intensity}\;\text{of}\;\left(\mathrm{MPI}/\text{BSA}\;\text{only}-\mathrm{MPI}/\text{BSA}\;\text{complexes}\right)\;}{\text{The}\;\mathrm{maximum}\;\text{fluorescence}\;\text{intensity}\;\text{of}\;\text{MPI}/\text{BSA}\;\text{only}}\times 100\%$$

The colloidal stability of EGCG, FEGCG, and FEGCG/Zn complexes was evaluated by incubating them with 0.9% NaCl at 37 °C for 2 h, and the FITC fluorescence was determined by multimode reader. The ROS/pH triggered MPI/BSA release from FEGCG/Zn complexes was evaluated by incubating them in medium with or without pH 5.0 PBS, 100 × 10^−6^ m H_2_O_2_ and both pH 5.0 and 100 × 10^−6^ m H_2_O_2_ for 24 h, respectively, and the FITC fluorescence was determined by multimode reader.

### Cytosolic Biomolecules Delivery

For cytosolic biomolecules delivery, Hep1‐6, MHCC‐97H, 4T1 cells were cultured and seeded in confocal dishes for confocal imaging or 12‐well plates for flow cytometry measuring. For DOX delivery, the cells were incubated with free DOX, FEGCG/6F‐DOX NPs, and FEGCG/Zn/6F‐DOX NPs (DOX 10 µg mL^−1^, w/w = 1) for 4 h. For siRNA delivery, the cells were incubated with free Cy3‐siRNA, EGCG/Cy3‐siRNA, FEGCG/Cy3‐siRNA, and FEGCG/Zn/Cy3‐siRNA polyplexes (siRNA 100 × 10^−9^ m, w/w = 25) for 4 h. For peptide/protein delivery, the cells were incubated with free MPI/BSA‐FITC, EGCG/MPI/BSA‐FITC, FEGCG/MPI/BSA‐FITC, and FEGCG/Zn/ MPI/BSA‐FITC (MPI‐FITC 10 µg mL^−1^, w/w = 1; BSA‐FITC 10 µg mL^−1^, w/w = 1) for 4 h. After incubation, the cells were washed with PBS and stained with DAPI before confocal microscopy or collected before flow cytometry.

### Preparation and Characterization of FEGCG/Zn/siRNA/Erythrocyte System

The erythrocytes were isolated from the blood of healthy C57BL/6 mice according to a standard procedure. All animals used in this study were approved by the Animal Care and Use Committee of the Affiliated Drum Tower Hospital of Nanjing University Medical School. Briefly, C57BL/6 female mice were sacrificed and the whole blood was collected from inferior vena cava. After that, the blood was centrifuged to remove the plasma, platelets, and white blood cells. The remaining cells were washed using ice cold 1× phosphate buffered saline (PBS, pH 7.4) buffer and centrifuged to obtain erythrocytes.

FEGCG/Zn/siRNA polyplexes were prepared as described above (1 mg kg^−1^ siRNA, w/w = 10). The attachment of FEGCG/Zn/siRNA polyplexes on erythrocytes could occur after mixing 100 µL FEGCG/Zn/siRNA polyplexes with 100 µL of cell suspensions. After 10 min, the hybridized FEGCG/Zn/siRNA/erythrocyte system was washed with PBS buffer for three times to remove the unattached polyplexes. Confocal microscopy was used to demonstrate the successful formation of FAM‐siRNA polyplexes on erythrocytes.

### Biodistribution Study

For biodistribution study in healthy mice, female C57BL/6 mice (six weeks old) were randomly divided into three groups of FEGCG/Zn/Cy5‐siRNA polyplexes, EGCG/Zn/siRNA/erythrocyte system, and FEGCG/Zn/siRNA/erythrocyte system (*n* = 3). At 0.5, 6, and 24 h after the above treatments (1 mg kg^−1^ siRNA, 10 mg kg^−1^ carrier, v/v = 1 of erythrocytes/polyplexes) injection by i.v., the animals were sacrificed and the major organs including heart, liver, spleen, lung, and kidneys were excised for imaging and quantifying with Caliper IVIS image system (excitation at 630 nm, emission at 680 nm). At 0.5 h, the excised lung tissues of different treatments were performed for immunofluorescence staining with Alexa Fluor 488 conjugated anti‐CD31 antibody.

For biodistribution study in Hep1‐6 tumor‐bearing mice, the tumor model was first established by subcutaneous injection the mixture of Hep1‐6 cells (5 × 10^6^) and matrigel. When the tumor allowed growing to ≈100 mm^3^, the female C57BL/6 mice were randomly divided into three groups as described above. At 1 and 12 h postinjection, the animals were imaged and the major organs and tumor were excised for imaging and quantifying.

### Antitumor Efficacy and Immune Activation Mechanism Study

The Hep1‐6 tumor‐bearing mice were established as described above. When the tumor allowed growing to ≈100 mm^3^, the female C57BL/6 mice were randomly divided into four groups (*n* = 6) of saline, FEGCG/Zn/siNC polyplexes, FEGCG/Zn/siPD‐L1 polyplexes, and FEGCG/Zn/siPD‐L1/erythrocyte system (1 mg kg^−1^ siRNA, 10 mg kg^−1^ carrier, v/v = 1 of erythrocytes/polyplexes). The treatment was performed on day 1, 3, 5, 7, and 9 by i.v. injection, and the tumor volume and body weight of the mice were recorded every other day. Tumor volume (mm^3^) = length × width^2^/2. On day 15, all mice were sacrificed and the tumor were collected for weight recorded, Tunel staining and PD‐L1 immunofluorescence staining according to a standard procedure.

For exploring the mechanism of enhanced immunotherapy, the blood was collected and the secreted cytokines of TNF‐*α* and IFN‐*γ* were determined by ELISA kits. For characterization of infiltrated CD8^+^ T cells, the lymphocytes were purified from tumor cells by gradient Percoll solutions with 80% and 40% concentration (GE Healthcare, Uppsala, Sweden). The amount of CD8^+^ T cells was detected by staining the lymphocytes with anti‐CD45‐APC (BL), anti‐CD3‐PE (BD), and anti‐CD8‐BV510 (BD) antibodies and analyzed by FCM (BD Biosciences, CA, USA). For characterization of exhausted CD8^+^ T cells, the exhausted CD8^+^ T cells were further purified from lymphocytes by the EasySep Mouse CD8 Positive Selection Kit (STEMCELL, Vancouver, Canada). The state of exhausted CD8^+^ T cells were detected by staining collected CD8^+^ T cells with anti‐CD8‐BV510 (BD), anti‐PD‐1‐PE‐Cy7 (eB), and anti‐Tim3‐PE (BL) antibodies according to a standard staining protocol and analyzed by FCM.

### Biosafety Study

For testing the potential toxicity, female C57BL/6 mice (six weeks old) were randomly divided into four groups as the same with the antitumor study. The treatments were administrated with saline, FEGCG/Zn/siNC polyplexes, FEGCG/Zn/siPD‐L1 polyplexes, and FEGCG/Zn/siPD‐L1/erythrocyte system for five times every other day. 24 h after the last injection, the mice were sacrificed and the major organs were collected for H&E staining. The blood was collected for alanine aminotransferase (ALT), aspartate aminotransferase (AST) determination, and blood analysis of WBCs, lymphocytes, neutrophils, monocytes, and platelets.

### Statistical Analysis

All statistical analyses were performed with GraphPad Prism 6 software. The value was expressed as means ± SD. The sample size (*n*) for each analysis is displayed in figure legends. An unpaired two‐tailed Student's *t*‐test was used for comparison of difference between two groups. A one‐way ANOVA test followed by Turkey's multiple comparisons was used for comparison of differences between more than two groups. The difference between the data sets was considered statistically significant at *P < 0.05, **P < 0.01.

## Conflict of Interest

The authors declare no conflict of interest.

## Supporting information

Supporting Information

## Data Availability

The data that support the findings of this study are available in the supplementary material of this article.
