# Supplementary material for: Engineered EGCG‐Containing Biomimetic Nanoassemblies as Effective Delivery Platform for Enhanced Cancer Therapy
Source: Adv Sci (Weinh). 2022 Mar 25;9(15):2105894. doi: 10.1002/advs.202105894 (PMC9131592; doi:10.1002/advs.202105894)
Supplement: Supplementary file 1 — Supporting Information [file ADVS-9-2105894-s001.pdf]

Supplementary Information

Engineered EGCG-Containing Biomimetic Nanoassemblies as Effective Delivery Platform for Enhanced Cancer Therapy

Pengkai Wu[1,3 #], Haitian Zhang[1 #], Yin Yin[1,3 #], Meiling Sun[3], Shuai Mao[3], Huihui Chen[1], Yexuan Deng[3], Shuai Chen[4], Shuo Li [2*], Beicheng Sun[1,3*]

[1]Department of Hepatobiliary Surgery, Nanjing Drum Tower Hospital, Clinical College of Nanjing Medical University, Nanjing 210008, Jiangsu Province, P.R. China

[2]Department of Gastroenterology, The First Affiliated Hospital of Nanjing Medical University, Nanjing 210029, Jiangsu Province, P.R. China

[3]Department of Hepatobiliary Surgery, Nanjing Drum Tower Hospital, The Affiliated Hospital of Nanjing University Medical School, Nanjing 210008, Jiangsu Province, P.R. China

[4]MOE Key Laboratory of Model Animal for Disease Study, Department of Endocrinology, Nanjing Drum Tower Hospital, and Model Animal Research Center, School of Medicine, Nanjing University, Nanjing 210008

[#] These authors contributed equally to this work.

*To whom correspondence should be addressed:

Beicheng Sun, M.D., Ph.D. Department of Hepatobiliary Surgery, Nanjing Drum Tower Hospital, Clinical College of Nanjing Medical University, Nanjing 210008, Jiangsu Province, P.R. China. Tel: 86-25-83105892; Email: sunbc@nju.edu.cn.

Shuo Li, MD, PhD Department of Gastroenterology, The First Affiliated Hospital of Nanjing Medical University, Nanjing 210029, Jiangsu Province, P.R. China. Tel:86-25-68306731; Email: shuoli@njmu.edu.cn

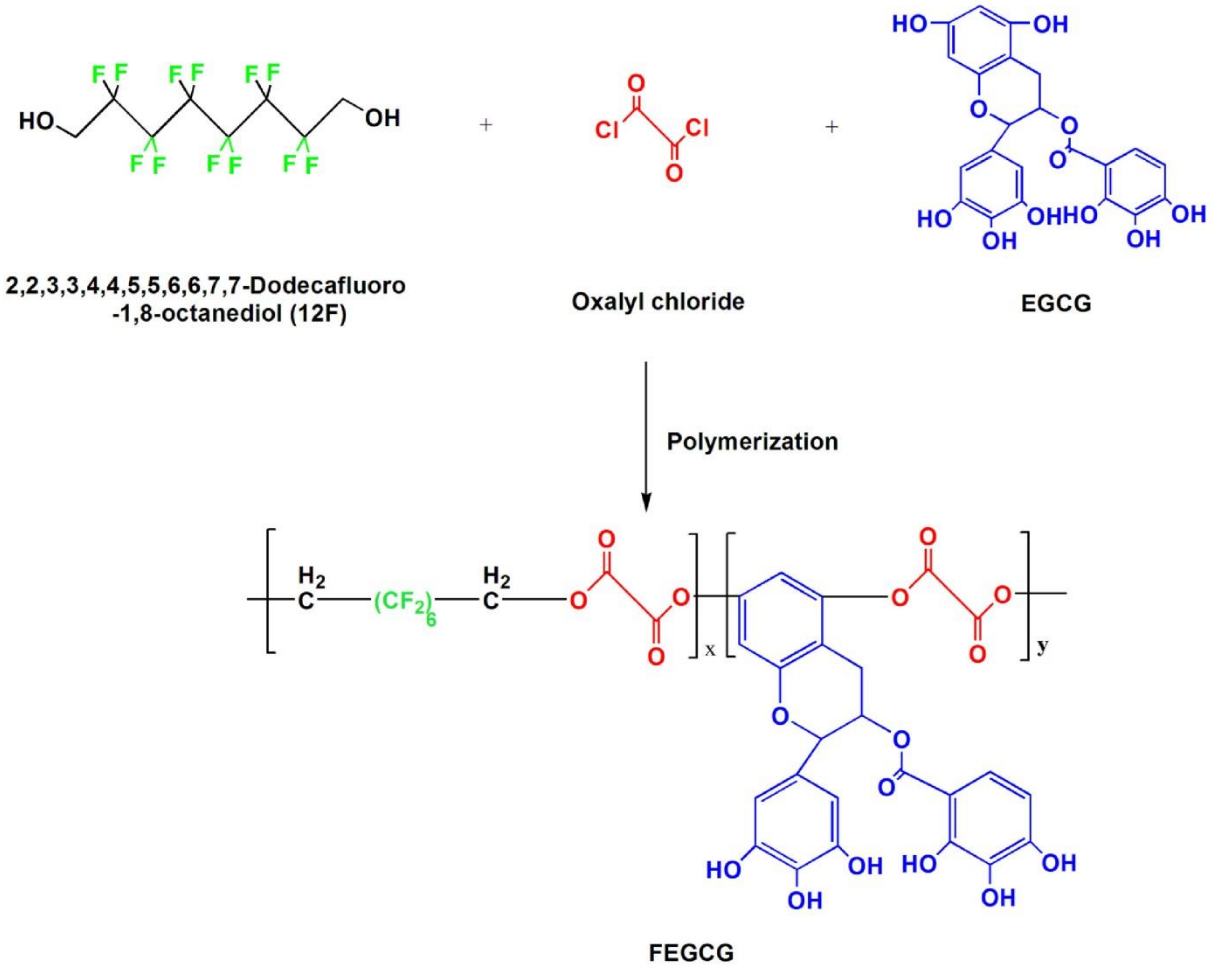

Figure S1. The synthesis route of FEGCG.

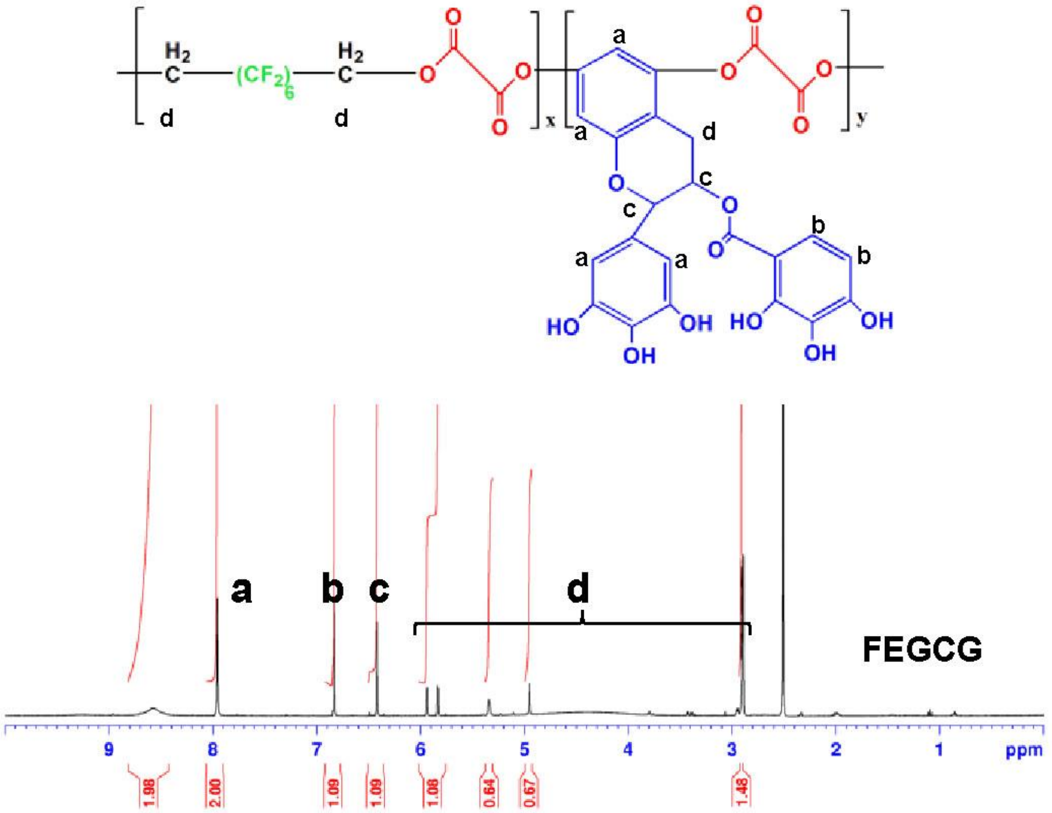

Figure S2. ^{1}H-NMR and ^{19}F-NMR spectrums of FEGCG in DMSO-d6.

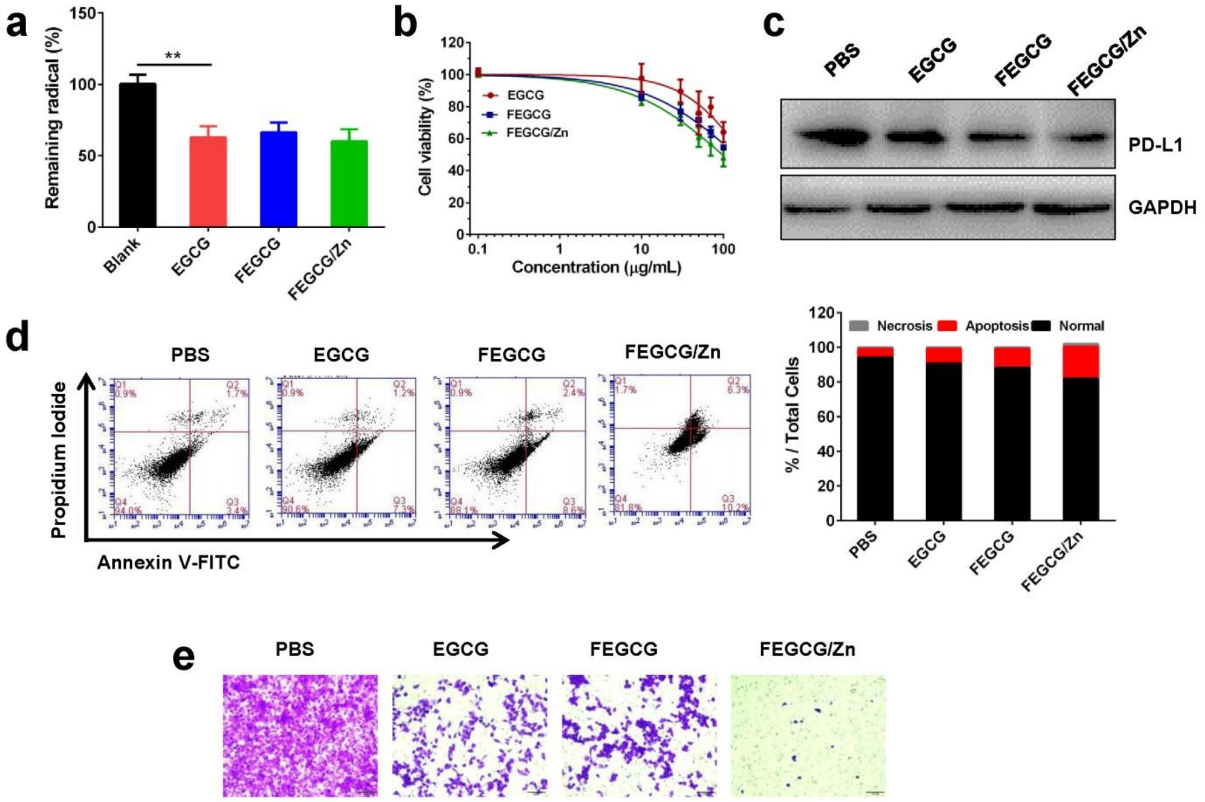

Figure S3. The free radical scavenging ability (n=3) (a), cytotoxicity (n=3) (b), PD-L1 expression (c), cell apoptosis (d) and cell migration ability (e) of EGCG, FEGCG and FEGCG/Zn in Hep1-6 cells. Data are presented as the means ± SD. Error bars represent the standard deviations of three separate measurements. **P < 0.05, **P < 0.01* by one-way ANOVA analysis followed by Turkey's multiple comparisons.

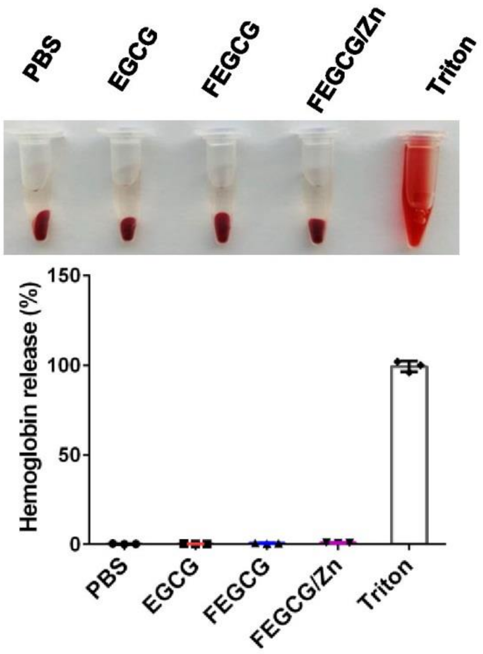

Figure S4. The hemolytic activity of EGCG, FEGCG and FEGCG/Zn on erythrocytes (n = 3). Data are presented as the means ± SD. Error bars represent the standard deviations of three separate measurements.

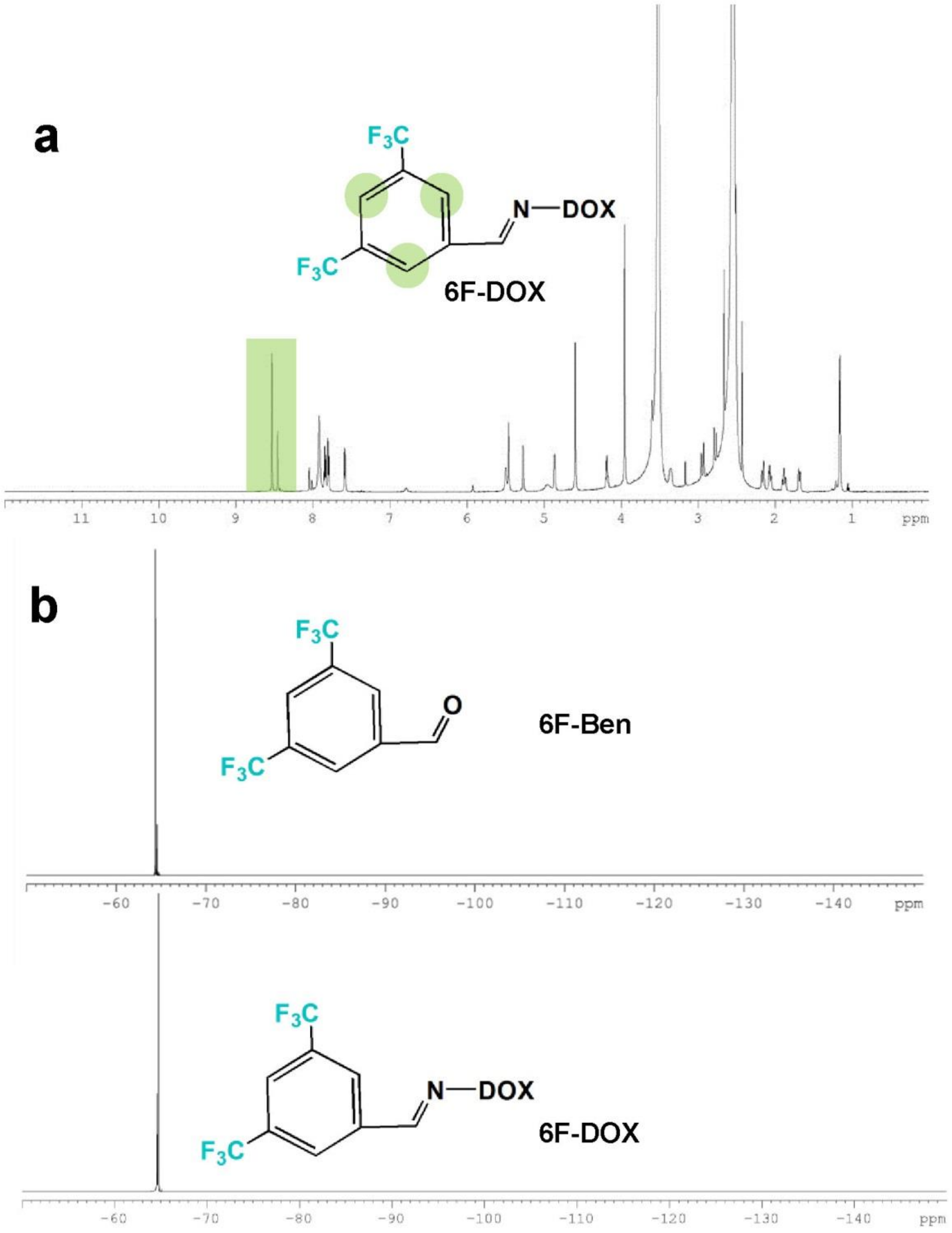

Figure S5. ^{1}H-NMR (a) and ^{19}F-NMR (b) spectrums of 6F-DOX in DMSO-d6.

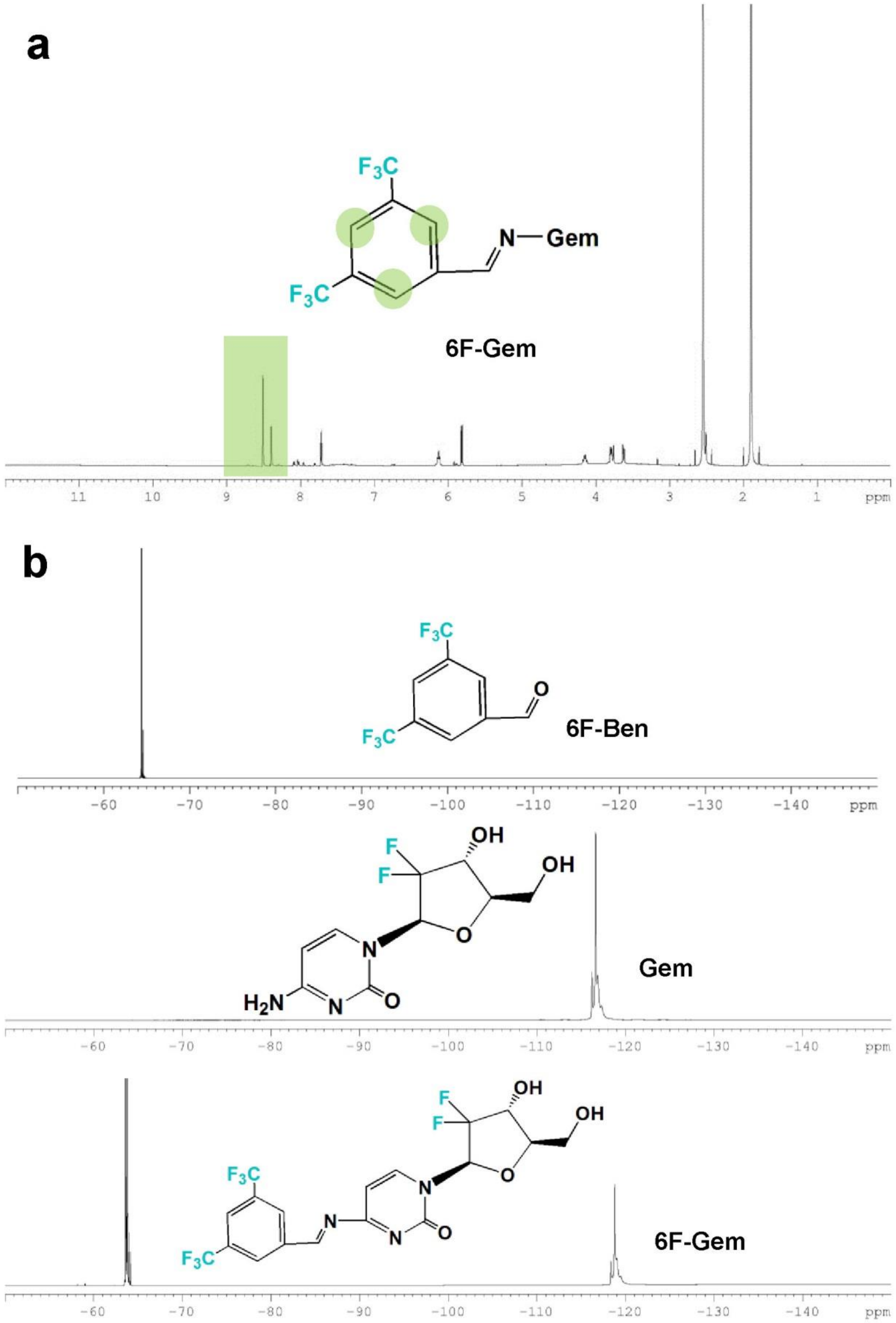

Figure S6. ^{1}H-NMR (a) and ^{19}F-NMR (b) spectrums of 6F-Gem in DMSO-d6.

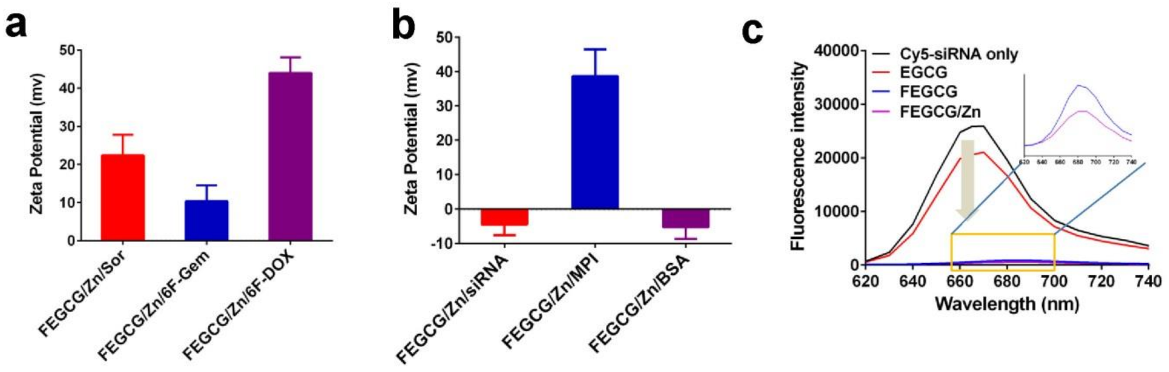

Figure S7. (a) The zeta potential of chemo-drugs loaded NPs (n = 3). (b) The zeta potential of siRNA polyplexes, MPI complexes and BSA complexes (n = 3). (c) siRNA loading efficiency determined by Cy5-siRNA fluorescence quenching assay. Data are presented as the means ± SD. Error bars represent the standard deviations of three separate measurements.

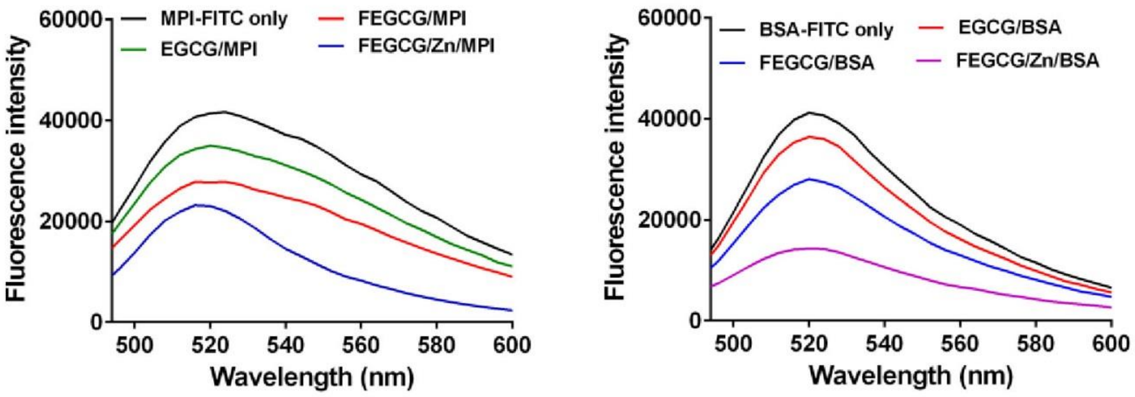

Figure S8. The related fluorescence spectrums of MPI only and different MPI complexes (a), and BSA only and different BSA complexes (b).

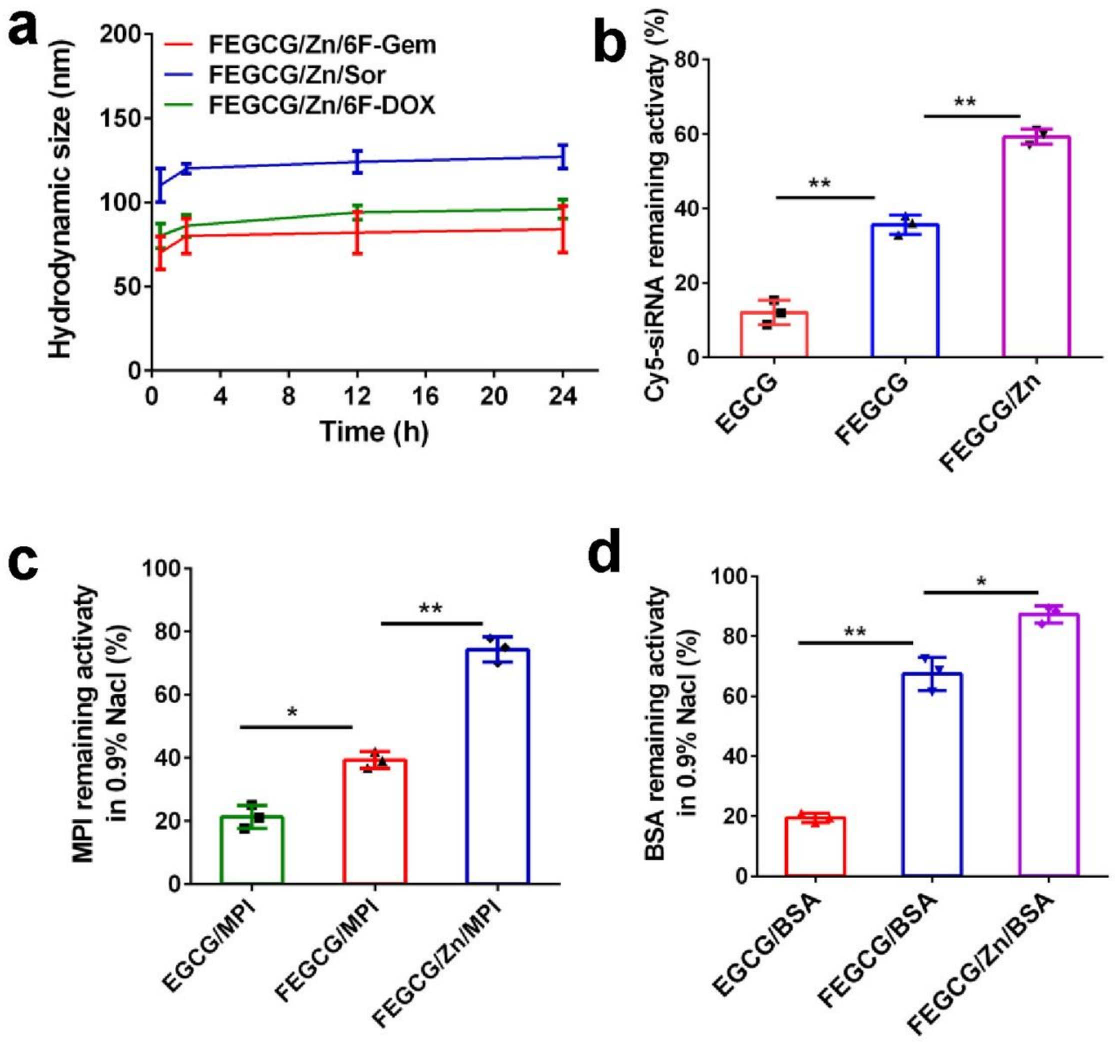

Figure S9. (a) The colloidal stability of chemo-drugs loaded NPs in PBS (n = 3). (b) The RNase stability of siRNA loaded polyplexes (n = 3). (c-d) The colloidal stability of MPI complexes and BSA complexes in 0.9% Nacl (n = 3). Data are presented as the means ± SD. Error bars represent the standard deviations of three separate measurements. *$*P < 0.05$, $**P < 0.01$* by one-way ANOVA analysis followed by Turkey's multiple comparisons.

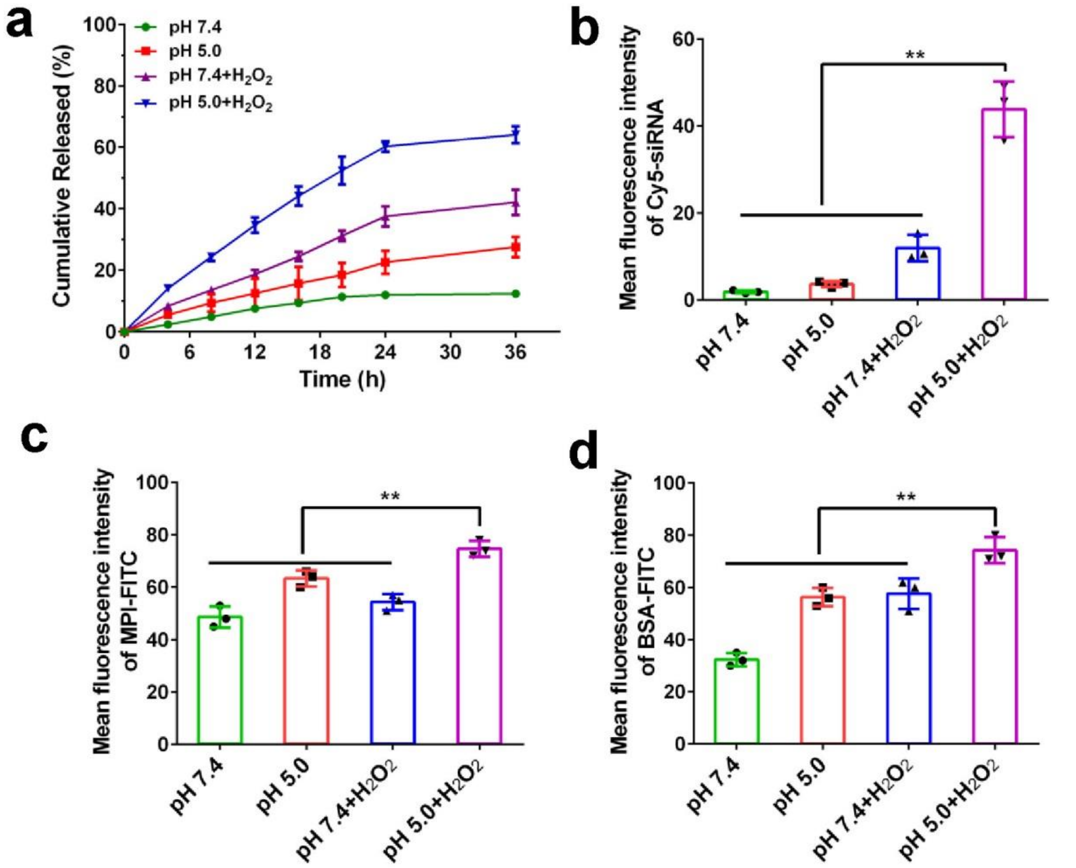

Figure S10. The *in vitro* sensitive release of FEGCG/Zn/6F-DOX NPs (a), FEGCG/Zn/siRNA polyplexes (b), FEGCG/Zn/MPI complexes (c) and FEGCG/Zn/BSA complexes (d) treated with H_2O_2 and different pH values (n = 3). Data are presented as the means ± SD. Error bars represent the standard deviations of three separate measurements. **P < 0.05, **P < 0.01* by one-way ANOVA analysis followed by Turkey's multiple comparisons.

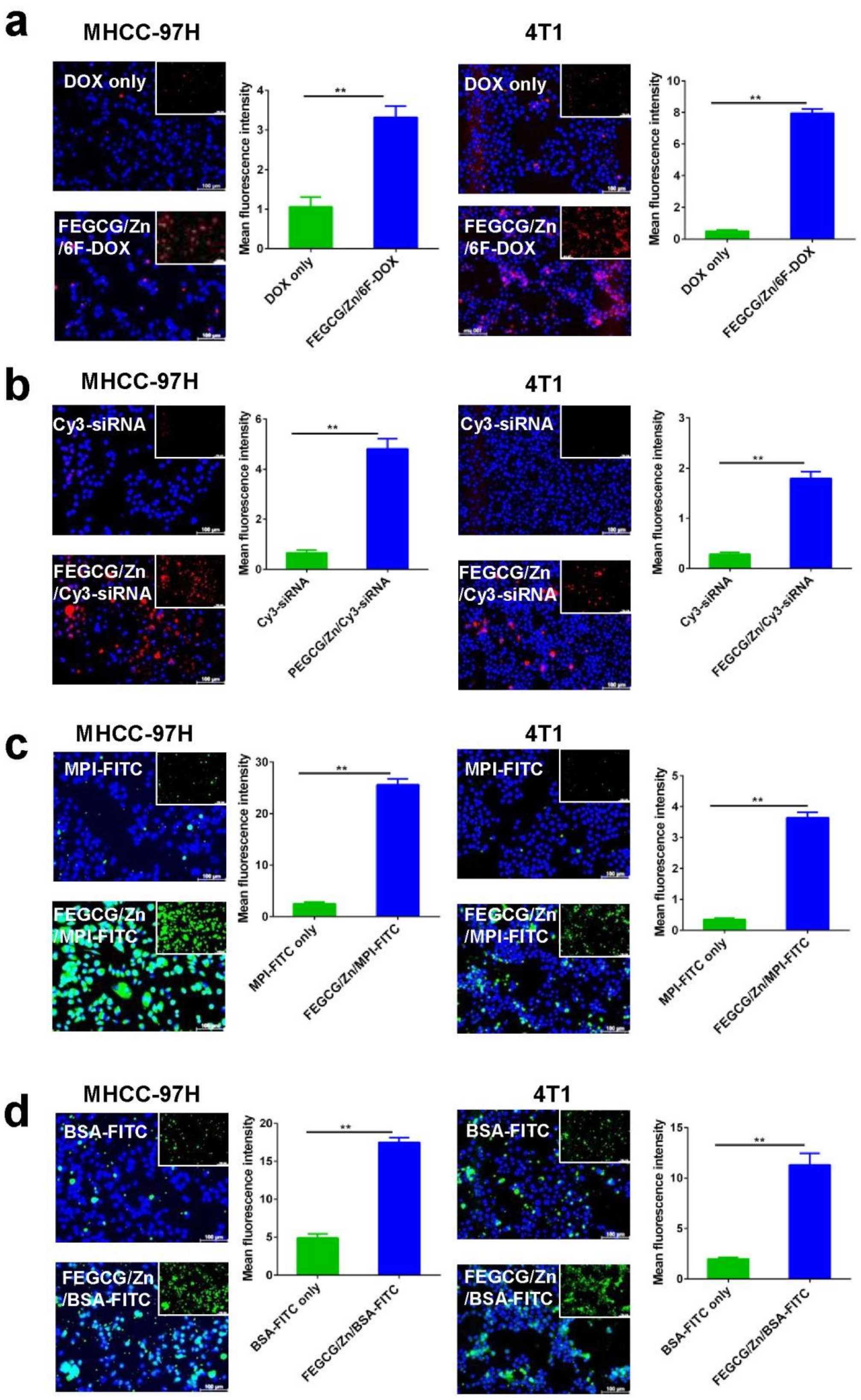

Figure S11. Confocal images and quantitative analysis of MHCC-97H and 4T1 cells treated with free DOX and FEGCG/Zn/6F-DOX NPs (a), free siRNA and FEGCG/Zn/siRNA

polyplexes (b), free MPI and FEGCG/Zn/MPI complexes (c), free BSA and FEGCG/Zn/BSA complexes (d) (n = 3). Data are presented as the means ± SD. Error bars represent the standard deviations of three separate measurements. *$P < 0.05$, **$P < 0.01$ by an unpaired two-tailed Student's t-test.

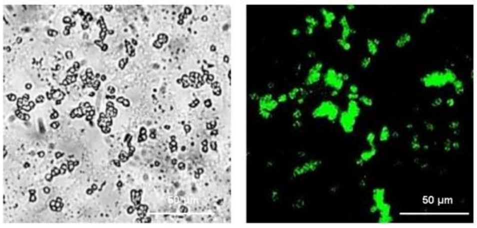

Figure S12. Confocal images of erythrocytes and FEGCG/Zn/FAM-siRNA/Erythrocyte system. Scale bar=50 μm.

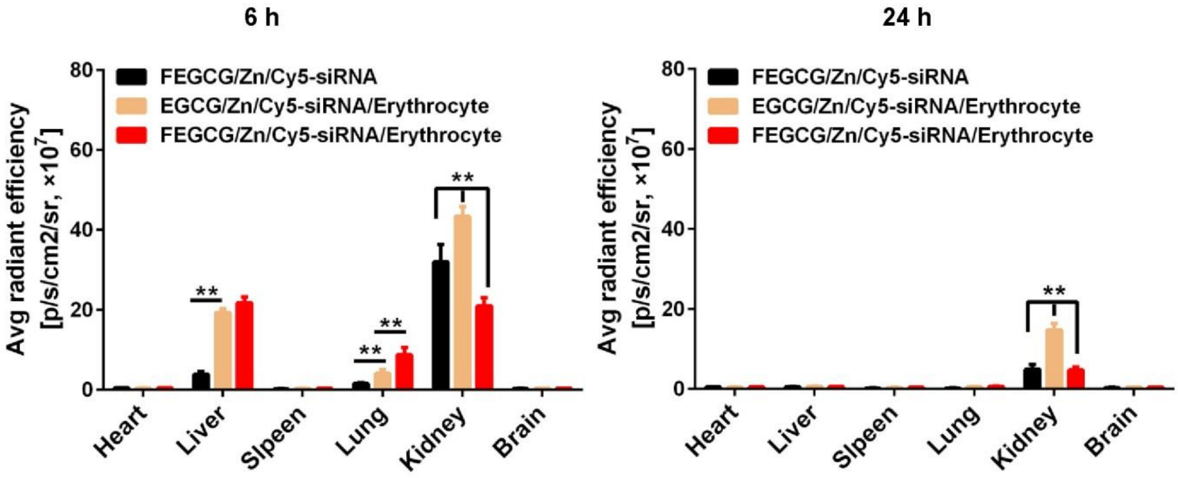

Figure S13. The biodistribution and quantitative analysis of FEGCG/Zn/Cy5-siRNA, EGCG/Zn/Cy5-siRNA/ Erythrocyte and FEGCG/Zn/Cy5-siRNA/Erythrocyte 6 h and 24 h after i.v. administration in healthy mice (n = 3). Data are presented as the means ± SD. Error bars represent the standard deviations of three separate measurements. *$P < 0.05$, **$P < 0.01$ by one-way ANOVA analysis followed by Turkey's multiple comparisons.

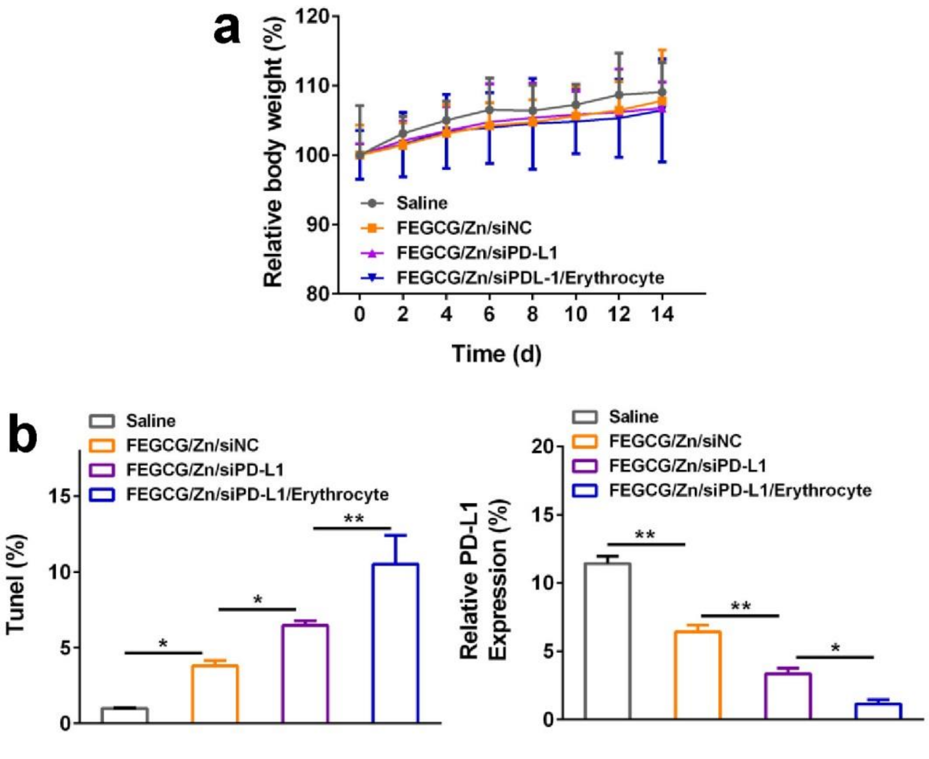

Figure S14. The body weights of each group during treatment period (n = 6) (a). (b) Quantitative analysis of Tunel staining and PD-L1 immunofluorescence staining in tumor tissues (n = 3). Data are presented as the means ± SD. Error bars represent the standard deviations of at least three separate measurements. **P < 0.05, **P < 0.01* by one-way ANOVA analysis followed by Turkey's multiple comparisons.

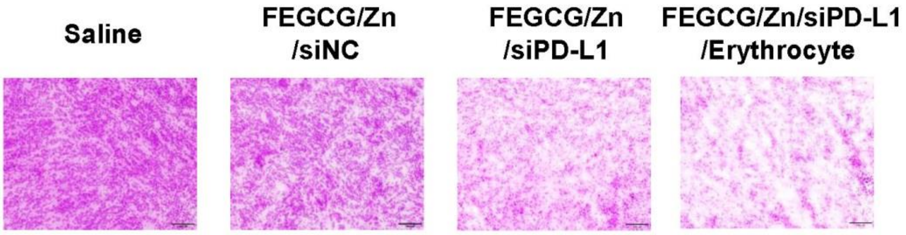

Figure S15. Representative images of H&E staining in tumor tissues after different treatments. Scale bar=100 μm.

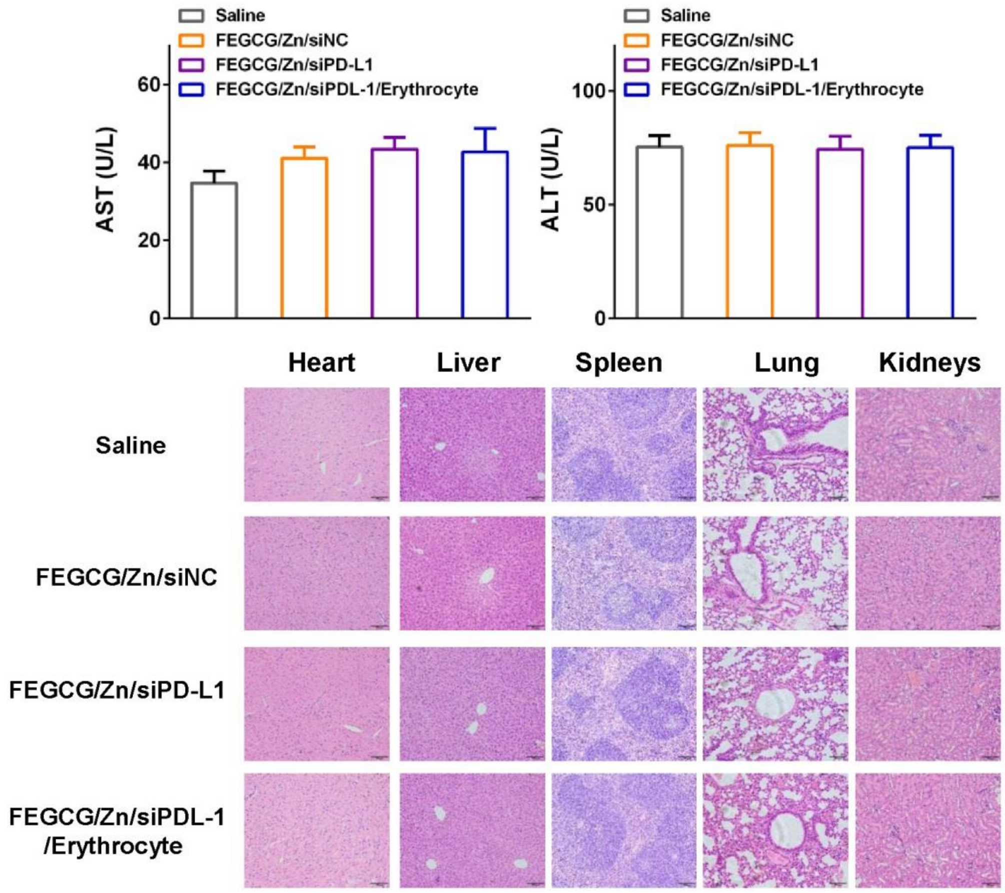

Figure S16. The serum biomarkers levels of AST and ALT (n = 3) (a-b), and the H&E staining of major organs (c) in healthy mice. Scale bar=200 μm. Data are presented as the means ± SD. Error bars represent the standard deviations of three separate measurements. **P < 0.05, **P < 0.01* by one-way ANOVA analysis followed by Turkey's multiple comparisons.

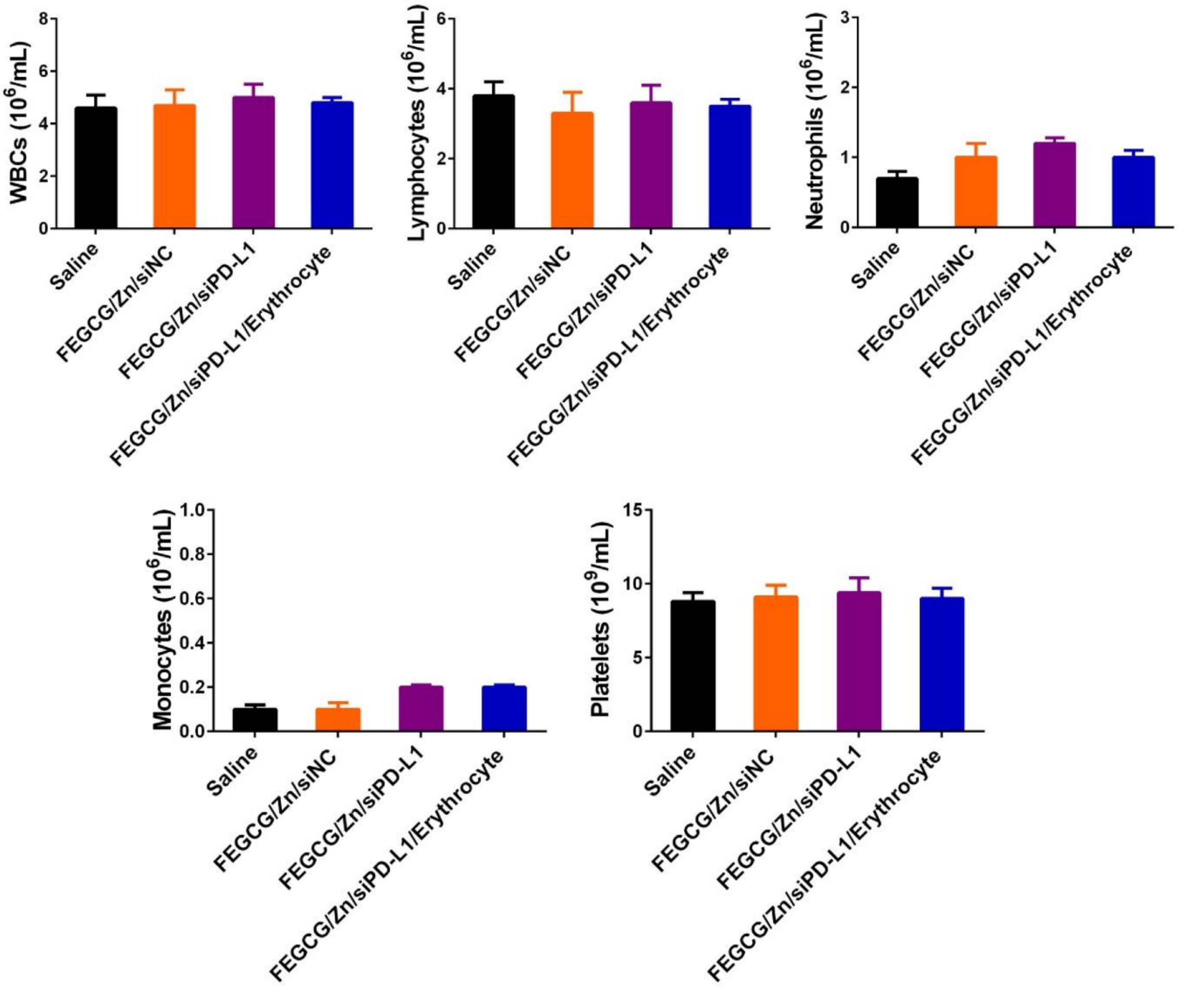

Figure S17. The blood analysis of WBCs, lymphocytes, neutrophils, monocytes and platelets after different treatments in healthy mice (n = 3). Data are presented as the means ± SD. Error bars represent the standard deviations of three separate measurements. *$*P < 0.05$, $**P < 0.01$* by one-way ANOVA analysis followed by Turkey's multiple comparisons.
